# Antimicrobial Resistance among Isolates Causing Invasive Pneumococcal Disease before and after Licensure of Heptavalent Conjugate Pneumococcal Vaccine

**DOI:** 10.1371/journal.pone.0005965

**Published:** 2009-06-18

**Authors:** Tom Theodore Karnezis, Ann Smith, Susan Whittier, Joseph Haddad, Lisa Saiman

**Affiliations:** 1 Department of Pediatrics, Columbia University Medical Center, New York, New York, United States of America; 2 Department of Pathology, Columbia University Medical Center, New York, New York, United States of America; 3 Division of Pediatric Otolaryngology, Columbia University Medical Center, New York, New York, United States of America; 4 Department of Epidemiology, NewYork-Presbyterian, Columbia University Medical Center, New York, New York, United States of America; University of Witwatersrand, South Africa

## Abstract

**Background:**

The impact of the pneumococcal conjugate vaccine (PCV-7) on antibiotic resistance among pneumococcal strains causing invasive pneumococcal disease (IPD) has varied in different locales in the United States. We assessed trends in IPD including trends for IPD caused by penicillin non-susceptible strains before and after licensure of PCV-7 and the impact of the 2008 susceptibility breakpoints for penicillin on the epidemiology of resistance.

**Methodology/Principal Findings:**

We performed a retrospective review of IPD cases at Morgan Stanley Children's Hospital of NewYork-Presbyterian, Columbia University Medical Center. Subjects were ≤18 years of age with *Streptococcus pneumoniae* isolated from sterile body sites from January 1995–December 2006. The rate of IPD from 1995–1999 versus 2002–2006 significantly decreased from 4.1 (CI_95_ 3.4, 4.8) to 1.7 (CI_95_ 1.3, 2.2) per 1,000 admissions. Using the breakpoints in place during the study period, the proportion of penicillin non-susceptible strains increased from 27% to 49% in the pre- vs. post-PCV-7 era, respectively (p = 0.001), although the rate of IPD caused by non-susceptible strains did not change from 1995–1999 (1.1 per 1,000 admissions, CI_95_ 0.8, 1.5) when compared with 2002–2006 (0.8 per 1,000 admissions, CI_95_ 0.6, 1.2). In the multivariate logistic regression model controlling for the effects of age, strains causing IPD in the post-PCV-7 era were significantly more likely to be penicillin non-susceptible compared with strains in the pre-PCV-7 era (OR 2.46, CI_95_ 1.37, 4.40). However, using the 2008 breakpoints for penicillin, only 8% of strains were non-susceptible in the post-PCV-7 era.

**Conclusions/Significance:**

To date, there are few reports that document an increase in the relative proportion of penicillin non-susceptible strains of pneumococci causing IPD following the introduction of PCV-7. Active surveillance of pneumococcal serotypes and antibiotic resistance using the new penicillin breakpoints is imperative to assess potential changes in the epidemiology of IPD.

## Introduction

Invasive pneumococcal disease (IPD) caused by strains non-susceptible to penicillin became increasingly prevalent during the mid-1990's. In 1998, the Center for Disease Control and Prevention reported that 24% of strains associated with IPD were non-susceptible to penicillin and that serotypes 6B, 9V, 14, 19F, and 23F accounted for 78% of penicillin non-susceptible strains [1]. In 2000, the heptavalent pneumococcal conjugate vaccine (PCV-7) was approved by the FDA to reduce the burden of pneumococcal disease, and in August 2000, the American Academy of Pediatrics recommended that all children younger than 2 years of age and high-risk children 2–5 years of age receive PCV-7 [2]. By 2006, 87% of American children 19–35 months of age had received 3 or more doses of PCV-7 [3] and numerous studies have documented a reduction in the incidence of IPD [4]–[6].

However, the impact of PCV-7 on the proportion of penicillin non-susceptible strains causing IPD has varied [5], [7]–[8]. Notably, these studies did not assess changes in the rates of invasive disease caused by such strains. In Utah, the proportion of penicillin non-susceptible strains decreased from 34% in the pre-PCV-7 era to 22% in the post-PCV-7 era [5]. In Dallas, no change in penicillin susceptibility was noted between 1999 and 2005 [7] and in Philadelphia, a 14% relative increase in the proportion of penicillin non-susceptible strains was observed following PCV-7 approval [8]. We assessed trends in hospital admissions for IPD before and after licensure of PCV-7 at our children's hospital in New York City. We also examined the epidemiology of antimicrobial susceptibility, including the impact of the 2008 susceptibility breakpoints for penicillin [9].

## Methods

### Study Design and Study Site

We reviewed cases of IPD admitted to the Morgan Stanley Children's Hospital of NewYork-Presbyterian, Columbia University Medical Center located in New York City from January 1, 1995 to December 31, 2006. The Institutional Review Board of Columbia University approved this study with a waiver of documentation of informed consent.

### Study Subjects and Study Isolates

The electronic data warehouse was queried for patients ≤18 years of age with a positive culture for *S. pneumoniae* isolated from blood, cerebrospinal fluid (CSF), pleural fluid, joint fluid, bone, and/or abscesses during the study period. If more than one culture from an individual patient was positive for *S. pneumoniae*, only the first was included in the analysis, and if both blood and CSF cultures were positive, the strain from CSF was included.

### Antimicrobial Susceptibilities

Antimicrobial susceptibilities as determined by the Clinical Laboratory and Standards Institute (formerly the National Committee on Clinical Laboratory Standards) in use during the study period [10] were compared to those introduced in 2008 [9]. During the study period, the breakpoints in use for penicillin for CSF and non-CSF isolates were comparable: susceptible ≤0.06, intermediate ≥0.1–1, and resistant ≥2 µg/ml. In 2008, the breakpoints were changed for both CSF isolates: susceptible ≤0.06 and resistant ≥0.12 µg/ml and for non-CSF isolates: susceptible ≤2, intermediate 4, and resistant ≥8 µg/ml. Breakpoints for 3^rd^ generation cephalosporins did not change; CSF isolates: susceptible ≤0.5, intermediate 1, and resistant ≥2 µg/ml and non-CSF isolates: susceptible ≤1, intermediate 2, and resistant ≥4 µg/ml.

### Statistical Analysis

Categorical variables were summarized as proportions. Pre- (1995–2000) and post- (2001–2006) PCV-7 variables were compared using the Chi-squared test and continuous variables were compared using the two-sample t-test. Multivariate logistic regression was used to estimate the rate change for penicillin susceptibility between the pre- and post-PCV-7 eras, controlling for age as a possible confounding variable. As PCV-7 was introduced in August 2000, 2000–2001 were considered to be transition years and thus the rate of IPD and the rate of IPD caused by penicillin non-susceptible strains was compared from 1995–1999 versus 2002–2006.

## Results

**Figure 1:**
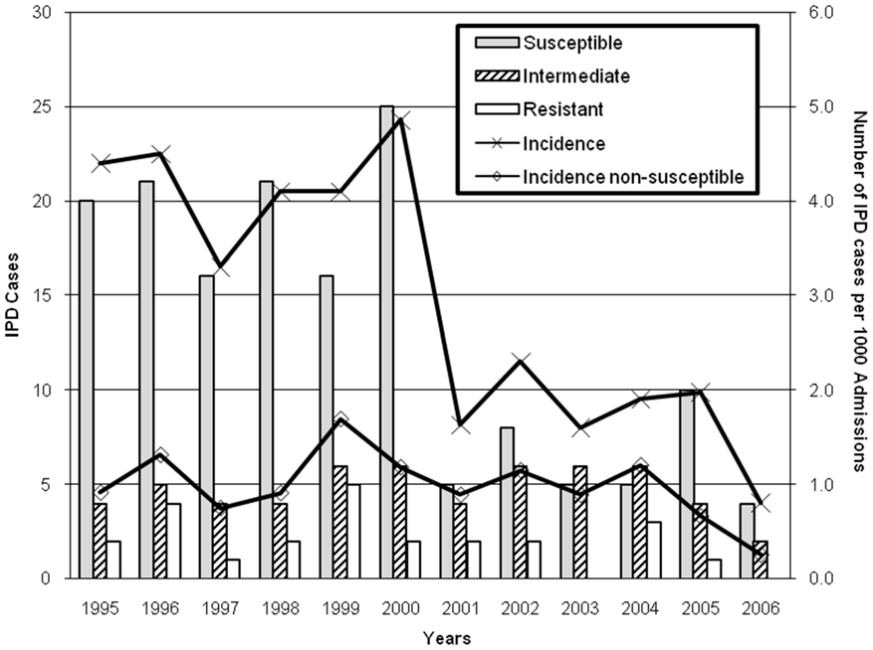
Invasive pneumococcal disease at a NYC Children's Hospital, 1995–2006. The number of cases of invasive pneumococcal disease (IPD) caused by penicillin resistant, intermediate, and susceptible strains of *S. pneumoniae* is shown (Left-hand Y axis). The rate of IPD per 1000 admissions and the rate of IPD caused by penicillin non-susceptible strains per 1000 admissions are shown (Right-hand Y-axis).

During the 12-year study period, 242 cases of IPD occurred of which 169 (70%) cases occurred from 1995–2000. Overall, bacteremia (n = 215) and meningitis (n = 17) were the most common diagnoses, and 56% of IPD cases occurred in boys. The rate of IPD from 1995–1999 vs. 2002–2006 significantly decreased (p<0.001) from 4.1 (CI_95_ 3.4, 4.8) to 1.7 (CI_95_ 1.3, 2.2) per 1,000 admissions as shown in the Figure 1.

Children with IPD were younger in the pre-PCV-7 era than children in the post-PCV-7 era (mean age 2.8 versus 4.6 years, respectively, p = 0.001). In the pre-PCV-7 era, children ≤2 years of age were less likely to be infected with non-susceptible strains (breakpoints in use during the study period) than older children (OR 0.4, CI_95_ 0.2, 0.8, p = 0.01) as 19 (20%) of 95 children ≤2 years of age versus 26 (37.7%) of 69 children >2 years of age were infected with non-susceptible strains. In the post-PCV-7 era, there was no association between age and non-susceptible strains as 15 (43%) of 35 children ≤2 years of age versus 21 (55%) of 38 children >2 years of age were infected with non-susceptible strains (p = 0.29).

As interpreted by the breakpoints in use during the study period, the MIC_50_ (median MIC) for penicillin in the pre-PCV-7 era was 0.06 µg/ml and in the post-PCV-7 era was 0.094 µg/ml, while the MIC_90_ (90^th^ percentile MIC) in the pre-PCV-7 era was 1.0 µg/ml and in the post-PCV-7 era was 2.0 µg/ml. In the pre-PCV-7 era, 45 (27%) of 164 tested strains were non-susceptible to penicillin (MIC ≥0.1 µg/ml) while in the post-PCV-7 era, 36 (49%) of 73 strains were non-susceptible (p = 0.001). In the multivariate logistic regression model controlling for the effects of age, strains of IPD in the post-PCV-7 era were significantly more likely to be penicillin non-susceptible than strains in the pre-PCV-7 era (OR 2.5, CI_95_ 1.4, 4.4, p = 0.003). However, the rate of IPD caused by non-susceptible strains did not change from 1995–1999 (1.1 per 1,000 admissions, CI_95_ 0.8, 1.5) when compared with 2002–2006 (0.8 per 1,000 admissions, CI_95_ 0.6, 1.2).

As expected, the breakpoints introduced in 2008 changed the epidemiology of penicillin resistance. A comparison of the proportion of penicillin non-susceptible strains using the previous and current breakpoints for non-CSF and CSF isolates from the pre- and post-PCV-7 era is shown in Table 1. Among the 220 non-CSF isolates from the study period, the proportion of penicillin-resistant strains would decrease from 10% (22/220) using previous breakpoints to 1.4% (3/220) using current breakpoints (p<0.001). Similarly, the proportion of intermediately susceptible non-CSF isolates would decrease from 24% (53/220) to 3% (6/220) [p<0.001]. In contrast, among the 17 CSF isolates from the study period, the proportion of resistant strains would not significantly change; there were 2 (12%) resistant strains using previous breakpoints vs. 6 (35%) resistant strains using current breakpoints (p = 0.106). Thus, in the post-PCV-7 era, there were only 4 non-CSF and 2 CSF isolates that were non-susceptible to penicillin using current breakpoints.

**Table 1:** Penicillin Susceptibility among Non-CSF and CSF Isolates in the Pre and Post PCV-7 Eras using Previous^1^ and Current^2^ Breakpoints*.

Breakpoint Interpretations	Non-CSF Isolates Pre-PCV-7 Era^3^		Non-CSF Isolates Post-PCV-7 Era		CSF Isolates Pre-PCV-7 Era		CSF Isolates Post-PCV-7 Era	
	Previous breakpoints	Current breakpoints	Previous breakpoints	Current breakpoints	Previous breakpoints	Current breakpoints	Previous breakpoints	Current breakpoints
**Susceptible**	110(73%)	146(97%)	35(51%)	65(94%)	9(69%)	9(69%)	2(50%)	2(50%)
**Intermediate**	27(18%)	3(2%)	26(38%)	3(4%)	2(15.5%)	0	2(50%)	0
**Resistant**	14(9%)	2(1%)	8(11%)	1(1%)	2(15.5%)	4(31%)	0	2(50%)
**Total**	151	151	69	69	13	13	4	4

* Abbreviations: 7-Valent Pneumococcal Conjugate Vaccine (PCV-7); Cerebrospinal fluid (CSF).

1 Breakpoints used prior to 2008.

2 Breakpoints introduced in 2008.

3 32 of the susceptible non-CSF isolates were processed using Kirby-Bauer and therefore did not have an MIC result and 5 non-CSF isolates from 1995 were missing susceptibility results.

There was no significant change in susceptibility to 3^rd^ generation cephalosporin agents (p = 0.66) in the pre- vs. post-PCV-7 eras; only 3.0% (4/132 tested strains) and 1.4% (1/70 tested strains) were non-susceptible to these agents, respectively. All tested strains (n = 208) were susceptible to vancomycin during the study period. In the post-PCV-7 era, all tested strains (n = 67) were susceptible to levofloxacin. In contrast, resistance to erythromycin increased from 6.7% (7/105 tested strains) in the pre-PCV-7 era to 29.6% (21/71 tested strains) in the post-PCV-7 era (p<0.001).

## Discussion

In our patient population, the incidence of IPD before and following PCV-7 licensure was similar to previous reports [4], [5], [7]. Prior studies have also documented an increase in the mean age of patients with IPD in the post-PCV-7 era [4], [5], [7]. However, as our children's hospital serves as a referral center, the rates presented are not population-based and our findings may not be generalizable to other clinical and geographic settings. Furthermore, we could not assess the efficacy of PCV-7 vaccine on actual cases of IPD.

To our knowledge, when compared with other studies performed throughout the U.S. (Table 2), this is one of the few reports to document an increase in the relative proportion of penicillin non-susceptible strains following the introduction of PCV-7. However, most previous reports have not addressed changes in the rate of IPD caused by non-susceptible pneumococcal strains and generally have not used the penicillin breakpoints recently introduced by the CLSI [9]. As we have shown, use of these new breakpoints would most likely decrease the proportion of non-susceptible strains isolated from non-CSF sources and increase the proportion of non-susceptible strains isolated from the CSF. Furthermore, we demonstrated that younger children were less likely to be infected with non-susceptible strains in the pre-PCV-7 era. We speculate that the shift to IPD in older children during the post-PCV-7 era could have influenced the penicillin non-susceptibility profile between the pre- and post-PCV-7 eras as older children could have had more exposure to antibiotics and thus be more likely to be infected with non-susceptible pneumococci.

**Table 2:** Comparison of Studies Describing the Impact of PCV-7 on Invasive Pneumococcal Disease in Children.

Ref	Study Sites (Years of Study)	Subjects Age	IPD Cases or Isolates (N)	Change in proportion of PCN non-susceptible strains pre vs. post PCV-7 licensure	Vaccine-Related Serotype change	Non-Vaccine-Related Serotype change
This study	New York, NY (1995–2006)	≤18 years	242 cases	22% increase (p = 0.001)	Not available	Not available
4	Selected sites in US^1^ (1999 vs 2004)	≤2 years^2^	858 isolates	81% decrease (CI_95_ (80%, 82%))	98% decrease (CI_95_ (96%, 99%))	150% increase
5	Intermountain Health Care System^3^ (1996–2000 vs 2001–2003)	<18 years	234 cases	12% decrease (p = 0.04)	23% decrease (p<0.001)	20% increase (p<0.001)
7	Dallas, TX (1999–2005)	≤17 years	398 cases	No change (p = 0.687)	Not available	64% increase (p<0.001)
8	Philadelphia, PA (1999–2000 vs 2001–2005)	≤17 years	188 cases bacteremia	14% increase (p = 0.019)	40% decrease	18% increase
11	Alaska (1995–2006)	<2 years	349 cases	6% decrease (p<0.001)	97% decrease	130% increase among Native children from 2001–2003 vs 2004–2006^4^
12	Cleveland, OH (1979–2004)	<5 years^2^	Not available	Not available	92% decrease (p<0.001)	64% decrease (p = 0.028)
13	Selected sites in US^1^ (1997–2004)	≤90 days	146 cases	75% decrease, PCV-7 serotypes (p<0.001) No change, non-PCV-7 serotypes	31% decrease	31% increase

* Abbreviations used in table: Reference (Ref) 7-Valent Pneumococcal Conjugate Vaccine (PCV-7); Invasive Pneumococcal Disease (IPD); Penicillin (PCN).

1 Included: San Francisco county, CA; Connecticut; 20 counties of Georgia, including Atlanta; Baltimore, Maryland; 7 counties surrounding St. Paul, Minnesota; 7 counties surrounding Rochester, New York; 3 counties surrounding Portland, Oregon; 5 urban counties, Tennessee.

2 This study presented data for all ages, but selected pediatric age strata were presented as indicated.

3 Included: Utah, Idaho, Montana, Wyoming, and parts of Arizona and Nevada.

4 No change in non-vaccine serotypes from 1995–2000 vs 2001–2003 among either Alaskan Native or Non-Native children.

While our findings may be due to pneumococcal serotype replacement or emergence of a new serotype in the post-PCV-7 era, such as been shown in diverse locations in the U.S., [Table 2] we were unable to assess this possibility because we did not systematically study serotypes.

Monitoring the burden of IPD in the United States is needed to continue to assess the efficacy of the PCV-7 vaccine as well as the efficacy of newer, more extensive valent PCVs. Surveillance must include the antimicrobial susceptibility and pneumococcal serotypes associated with IPD. While our results suggest the continued need for empiric treatment of penicillin non-susceptible strains, particularly if meningitis is suspected, epidemiologic trends for antimicrobial susceptibility should be studied with the new penicillin breakpoints.
